# A Contribution to the Surgery of the Spinal Cord

**Published:** 1890-06

**Authors:** 


*A Contribution to the Surgery of the Spinal Cord.* By W. THORBURN, B.S., M.D., F.R.C.S. Pp. 230. London: C. GRIFFIN & CO. 1889.

A young surgeon can scarcely do better work than this; namely, to collate all the clinical experience within his reach bearing on an imperfectly worked-out subject, and by the light of this, and full theoretical knowledge, try to formulate new conclusions or to confirm old beliefs. The author has fully succeeded in the task he has set himself; and has provided us with a most thorough and exhaustive work on spinal surgery. He is fully acquainted with all the latest researches on spinal physiology and pathology, and has most fully and carefully applied his knowledge to the numerous cases collected and recorded by him. We gladly acknowledge our indebtedness to the author for his valuable and suggestive treatise; and have great pleasure in recommending it to our readers.

